# Supplementary material for: Transient viral exposure drives functionally-coordinated humoral immune responses in HIV-1 post-treatment controllers
Source: Nat Commun. 2022 Apr 11;13:1944. doi: 10.1038/s41467-022-29511-1 (PMC9001681; doi:10.1038/s41467-022-29511-1)
Supplement: Supplementary file 1 — Supplementary Information [file 41467_2022_29511_MOESM1_ESM.pdf]

Supplementary information

Transient Viral Exposure Drives Functionally-Coordinated Humoral Immune Responses in HIV-1 Post-Treatment Controllers

Luis M. Molinos-Albert[1,2], Valérie Lorin[1,2], Valérie Monceaux[3], Sylvie Orr[4], Asma Essat[4], Jérémy Dufloo[5], Olivier Schwartz[5], Christine Rouzioux[6], Laurence Meyer[4], Laurent Hocqueloux[7], Asier Sáez-Cirión[3], Hugo Mouquet[1,2*]; ANRS VISCONTI Study Group.

[1]Laboratory of Humoral Immunology, Department of Immunology, Institut Pasteur, and [2]INSERM U1222, Paris, 75015, France
[3]HIV, Inflammation and Persistence Unit, Department of Virology, Institut Pasteur, Paris, 75015, France
[4]Centre de Recherche en Epidémiologie et Santé des Populations (CESP), Université Paris-Sud, Université Paris-Saclay, INSERM, Le Kremlin-Bicêtre, France
[5]Virus & Immunity Unit, Department of Virology, Institut Pasteur, and [6]CNRS URA3015, Paris, 75015, France
[6]Assistance Publique-Hôpitaux de Paris, Service de Microbiologie Clinique, Hôpital Necker-Enfants Malades, Paris, France
[7]Service des Maladies Infectieuses et Tropicales, CHR d'Orléans-La Source, Orléans, 45067, France

Supplementary table 1

Supplementary figures 1 - 9

Supplementary table 1. Clinical and immunovirological characteristics of post-treatment controllers (PTC, ANRS VISCONTI) and post-treatment non controllers (PTNC, ANRS CO6 PRIMO) included in the study

										pre-ART		pre-TI		Post-TI[1]					
	ID	Gender	Ethnicity	Age	HLA B35/53	ART regimen	ART initiation[2] (estimated day post-infection)	Time on ART (years)	PTC status/TI date (dd/mm/yy)	VL	CD4	VL	CD4	VL	CD4	Time Post-TI (years)	Time from ART (years)	% VL>50	VL test (N)
	090001	M	C	27	+	FTC-TDF-DAV/RTV->DAV/RTV	37	2.00	26/06/2012	4472974	185	40	441	40	498	5.0	7.0	0.0%	
	041001	M	C	54	-	SQV-DDI-NFV-D4T	30	2.40	25/07/2000	476100	351	200	715	20	829	17.2	19.6	0.0%	
	098004	M	C	67	+	LPV/RTV-3TC-AZT->LPV/RTV-3TC-AZT-ABC	87	3.00	13/04/2005	444304	419	50	856	40	756	12.6	15.6	0.0%	
**PTC**	180002	F	A	45	+	ABC-3TC-AZT-NFV->ABC-3TC-AZT	61	2.00	17/11/2003	6165950	955	20	906	20	704	13.0	15.0	0.0%	
**Stably**	063001	F	A	45	+	3TC-AZT-IDV/RTV->ABC-3TC-AZT	22	1.80	15/11/2001	2064	803	50	1354	20	1013	15.3	17.1	0.0%	
**aviremic**	056003	F	A	51	-	3TC-AZT-EFV->3TC-EFV-TDF	68	3.70	03/04/2006	2260000	471	50	1152	20	1474	11.9	15.6	0.0%	
**(sPTC)**	093001	F	C	38	-	3TC-AZT-LPV/RTV->3TC-D4T-LPV/RTV	28	1.40	01/07/2006	730000	393	50	793	40	903	13.3	14.7	0.0%	
	098003	M	C	42	-	LPV/RTV-FTC-TDF	54	1.00	20/02/2008	240000	302	40	786	40	427	8.2	9.2	0.0%	
	180001	M	C	51	+	AZT-DDI->3TC-AZT	81	6.67	15/02/2003	20893	416	20	1057	20	1019	13.9	20.6	0.0%	
	087001	M	C	32	+	TDF/FTC ATV/RTV->TDF/FTC/RPV	47	2.30	15/08/2015	28009	513	40	417	40	625	2.6	4.9	0.0%	
	200001[3]	M	C	51	+	3TC-AZT-EFV->3TC-AZT-DDI	56	1.00	22/10/2002	1070	397	50	523	2933	494	12.7	13.7	73.7%	38
	070001[3]	M	C	61	+	AZT-DDI->AZT-DDI-IDV	36	16.80	24/01/2013	1800	789	40	1675	5003	1540	2.1	18.9	53.8%	13
	216001[3]	M	C	47	-	3TC-AZT-NFV	33	3.20	24/10/2002	21533	593	40	824	86480	541	8.6	11.8	46.2%	26
	180003	F	C	51	-	3TC-AZT-RTV	0+147	7.10	13/10/2003	2400	NA	50	354	1040	375	14.3	21.4	55.0%	40
**PTC**	098001	M	C	69	-	D4T-IDV/RTV-DDI->3TC-AZT-LPV/RTV	19	1.90	07/03/2003	74700	682	50	583	40	392	8.7[4]	10.6	18.9%	37
**Virally**	005002	M	C	37	-	TDF/FTC DRV/RTV->ABC/3TC DRV/RTV	493	2.50	23/12/2016	182763	594	20	537	98	601	2.8	5.3	100.0%	3
**exposed**	063003	M	C	42	-	3TC-AZT-RTV->3TC-AZT	26	11.20	01/10/2007	137849	937	40	1018	20	945	10.5	21.7	22.2%	9
**(ePTC)**	038001	F	C	62	+	AZT/3TC	584	2.20	15/06/2001	53000	550	200	810	50	848	15.9	18.1	16.1%	31
	005001	M	C	38	-	TDF/FTC FosA/RTV	94	10.00	01/06/2015	309820	191	20	1318	20	449	3.5	13.5	16.7%	6
	056002	F	A	71	+	D4T-IDV-3TC->D4T-LPV/RTV-3TC	43	6.10	10/03/2004	10500	738	50	1166	40	1210	12.9	19.0	6.3%	16
	073001	F	A	20	-	AZT-DDI-3TC	0+93	5.50	15/10/2002	2170000	2984	50	669	20	600	14.4	19.9	3.2%	31
	084001	M	C	49	+	3TC-AZT-IDV->3TC-AZT-IDV/RTV	34	1.00	18/08/2000	12302200	371	50	1896	40	1331	18.0	19.0	2.9%	35
	130102	M	C	50	-	3TC-AZT-IDV->3TC-AZT-NFV	26	4.48	04/02/2002	2999589	329	10	329	5240	717	1.6	6.0		
	130105[5]	M	C	42	-	3TC-AZT-EFV->3TC-AZT-DDI	37	4.81	16/05/2005	1470000	256	10	656	10	489	7.6	12.4		
	130207	M	C	29	-	3TC-AZT-NVP->3TC-AZT-EFV	94	2.91	07/02/2002	3700	734	10	806	168	NA	1.1	4.0		
	130210	F	C	43	-	3TC-D4T-NFV->3TC-AZT-DDI->3TC-DDI-TDF	36	2.89	01/11/2003	2180000	293	10	606	35000	380	1.3	4.1		
	600101	M	C	21	+	3TC-AZT-NFV->3TC-AZT-ABC	41	3.29	28/04/2003	115468	655	10	1075	7010	891	0.7	4.0		
	680112	M	C	31	-	3TC-AZT-IdR->3TC-AZT->3TC-AZT-ABC	63	3.06	27/02/2004	500000	1041	10	1567	37618	806	0.9	4.0		
	750202	M	C	42	-	D4T-DDI-NFV	36	3.82	25/10/2002	53320	701	10	700	890	852	5.0	8.8		
	750401	F	C	37	-	3TC-AZT-IDV->3TC-AZT->3TC-AZT-NFV->3TC-AZT-ABC	83	4.34	26/06/2001	7940	571	10	1049	206	945	0.7	5.0		
	750407	M	C	30	+	3TC-AZT-LpR	41	0.48	18/12/2003	1900000	309	10	721	45300	330	1.5	2.0		
	750702	M	C	37	-	3TC-D4T-SQV->3TC-D4T-NVP	40	2.96	10/10/2000	5012	612	10	700	13339	676	1.0	4.0		
**PTNC**	750706	M	C	28	-	D4T-DDI	83	3.84	21/01/2003	220	631	10	686	18485	522	1.1	5.0		
	750712	M	C	31	-	3TC-AZT-NFV->3TC-AZT-NVP	51	3.29	30/04/2003	58196	620	10	930	3313	496	3.7	7.0		
	750804	M	C	25	-	3TC-AZT-IDV->3TC-AZT-NVP	39	4.51	12/09/2003	272859	521	10	1149	9600	744	0.5	5.0		
	751101	M	C	36	-	3TC-AZT-IDV->3TC-AZT-NVP	37	6.08	15/10/2003	3162300	230	10	649	34700	483	1.3	7.4		
	751102	M	C	31	-	3TC-D4T-IDV->3TC-AZT-ABC->3TC-AZT-EFV->3TC-ABC-DDI	100	5.13	20/01/2003	16000	632	10	1138	4400	942	1.9	7.0		
	751104	M	C	31	+	3TC-AZT-NFV->D4T-DDI-EFV	26	4.27	09/01/2003	2000000	451	10	731	105000	420	2.0	6.3		
	780102	M	C	38	-	3TC-AZT-NFV->3TC-AZT-NVP	85	4.93	13/06/2003	35526	705	10	780	9324	450	0.6	5.5		
	910104	M	C	32	+	3TC-ABC-AZT	27	2.71	15/04/2002	83000	527	10	488	112143	446	0.3	3.0		
	930210	M	C	37	-	3TC-AZT-FpR->3TC-AZT-LpR	34	2.49	28/11/2007	464487	772	10	1340	59140	854	0.5	3.0		
	940216	M	C	51	+	3TC-AZT-LpR	76	0.48	20/03/2007	124000	412	10	599	35672	361	1.0	1.5		
	250108	M	C	53	+	3TC-AZT-LpR->3TC-LpR-D4T	51	1.40	01/03/2005	2600000	958	NA	NA	226000	622	0.7	2.1		

F, female; M, male; A, African; C, Caucasian; TI, treatment interruption; VL, viral loads, copies/mL plasma; CD4, cells/μl; NA, not available.

[1]Last follow up for PTC; before ART ressumtion for PTNC

[2]For PTC 005001 and 056002, delay between diagnosis and ART initiation are indicated; for PTC 180003 and 073001, 0 and the second value indicate the initiation of transient post-exposure prophylaxis and long-term ART, respectively

[3]ePTC rebounders who resumed to ART by the time or before the analysis

[4]ART ressumption due to development of lymphoma

[5]under cART at analysis. Viral rebound 10 months post-TI (VL 641 c/mL, CD4 count 380); VL peak 26 months post-TI (1670 c/mL, CD4 count 359)

3TC Lamivudine ABC Abacavir AZT Zidovudine D4T Stavudine DaR Darunavir-Ritonavir DDI Didanosine EFV Efavirenz FpR Fosamprenavir-Ritonavir FTC Emtricitabine IdR Indinavir-Ritonavir IDV Indinavir LpR Lopinavir-Ritonavir NFV Nelfinavir NVP Nevirapine RTV Ritonavir SqR Saquinavir-Ritonavir SQV Saquinavir TDF Tenofovir

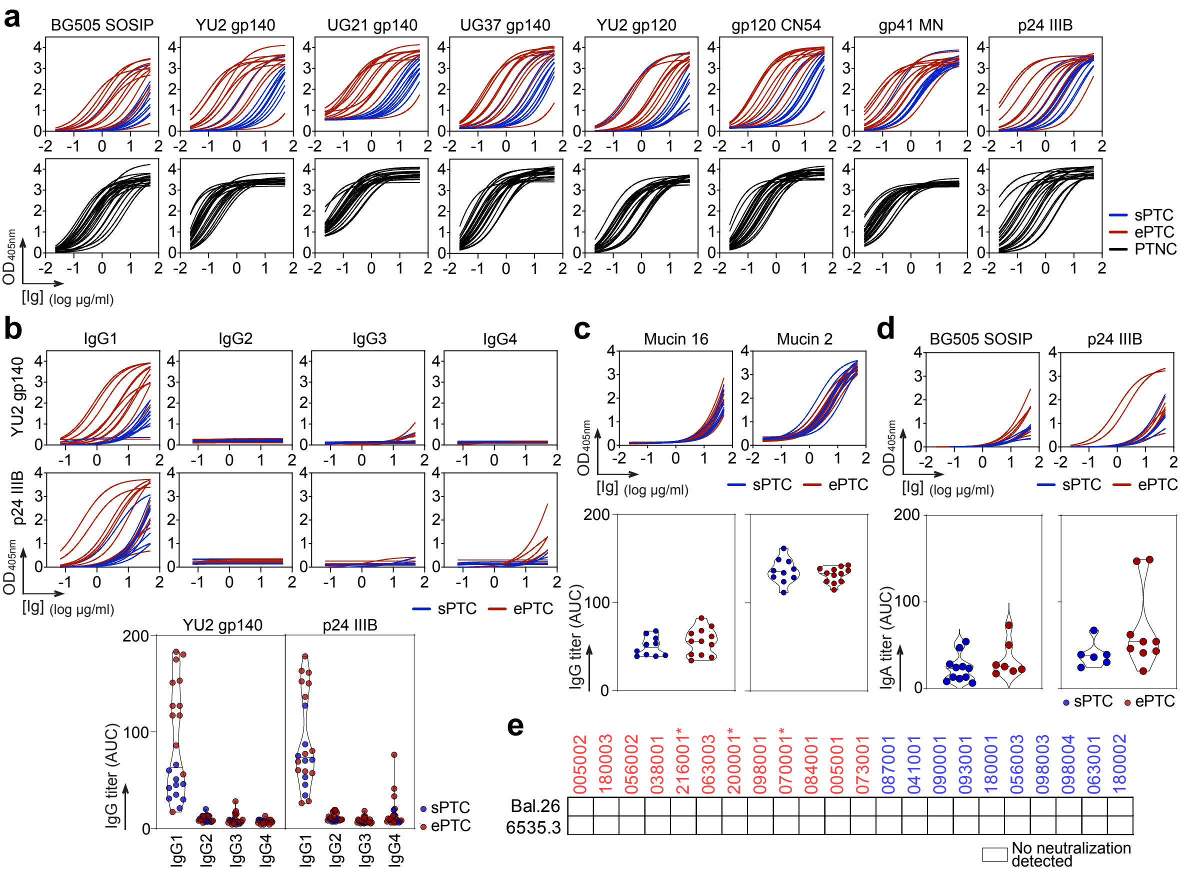

Supplementary Fig. 1. Serum antibody binding to HIV-1 and mucin proteins in PTC and PTNC. (**a**) ELISA graphs comparing the binding of serum IgG antibodies purified from PTC (top, n=22) and PTNC (bottom, n=21) to selected HIV-1 antigens. (**b**) ELISA graphs showing the subclass reactivity of serum IgG antibodies purified from PTC (n=22) against HIV-1 Env gp140-F and p24 proteins (left); corresponding area under the curve (AUC) values are presented in the violin plots (bottom). Each dot corresponds to a donor. (**c**) Same as in (b) but for the binding of serum IgG antibodies purified from PTC (n=22) against human mucin isoforms 2 and 16. (**d**) Same as in (b) but for the binding of serum IgA antibodies purified from PTC (n=18) against HIV-1 Env SOSIP and p24 proteins. (**e**) Heatmap comparing the IgA seroneutralizing activity in PTC against two tier-1 viruses. White color indicates no neutralization detected with purified serum IgA antibodies at a concentration of 250 µg/ml. The asterisks indicate ePTC rebounders. Source data are provided as a Source Data file.

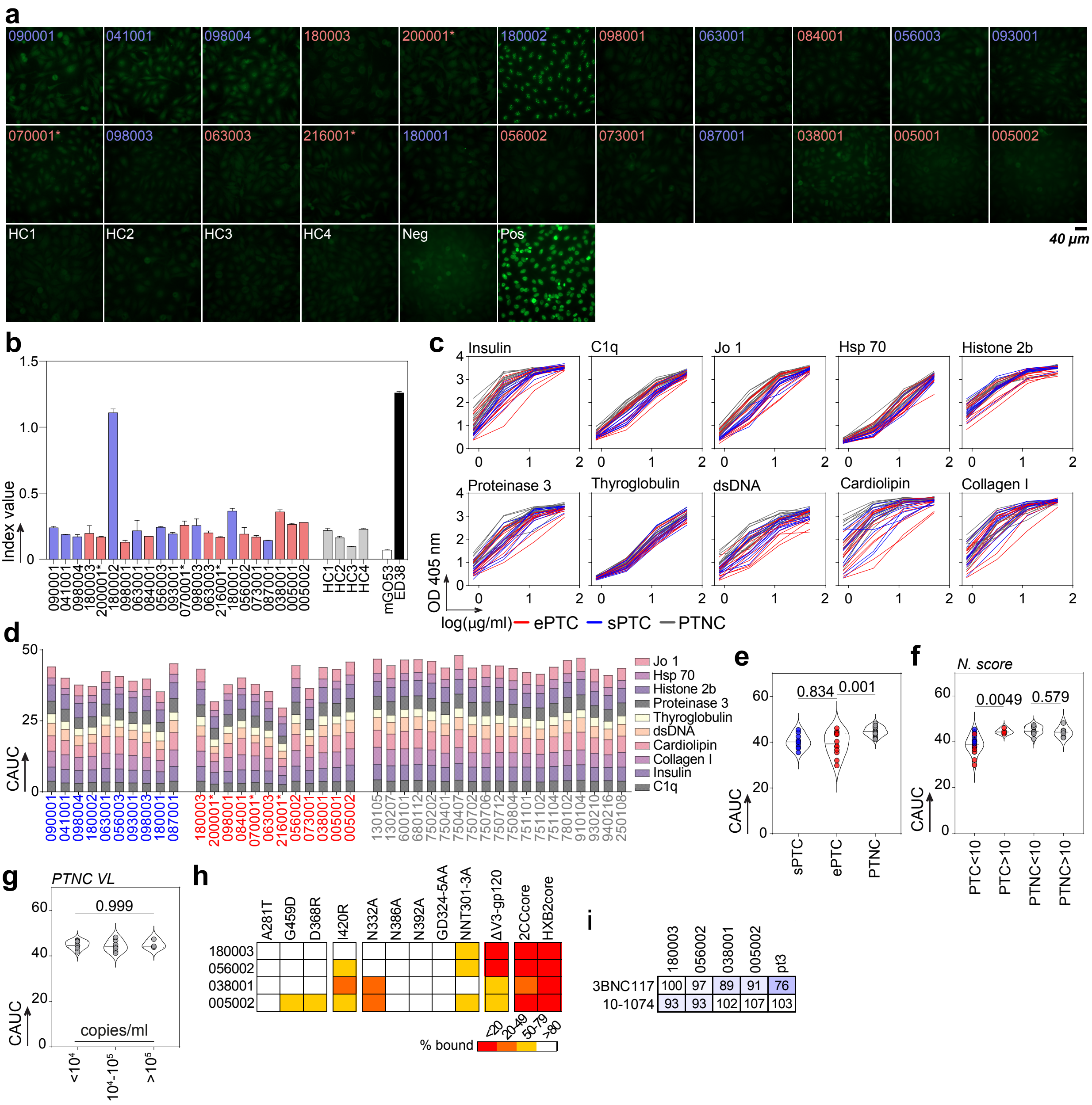

Supplementary Fig. 2. Serum IgG autoreactivity and epitope mapping in PTC. (**a**) Microscopic images showing the reactivity of serum IgG antibodies purified from PTC to HEp2-expressing self-antigens assayed by indirect immunofluorescence assay. The positive (Pos) and negative (Neg) controls of the kit were included in the experiment. Purified serum IgGs from seronegative healthy donors (HC1 to HC4) were also tested for comparison. The scale bars represent 40 µm. The images are representatives of two independent experiments. (**b**) Bar graph shows the HEp-2 reactivity as measured by ELISA. Means ± SD of duplicate values are shown. (**c**) ELISA graphs showing the binding of serum IgG antibodies purified from PTC (n=22) and PTNC (n=19) to selected self-antigens. (**d**) Bar graph showing for each donor, the global reactivity against all self-antigens expressed as the cumulative area under the curve (CAUC). (**e**) Violin plot comparing the reactivity to self-antigens as CAUC between sPTC (blue, n=10), ePTC (red, n=12) and PTNC (grey, n=19). (**f**) Same as in (e) but segregating PTC and PTNC according to the neutralization (N.) scores < or > to a value of 10. (**g**) Same as in (e) but segregating PTNC according to the viral loads (VL) of $<10^4$ (n=9), 10^4-10^5 (n=7) and $>10^5$ (n=3) copies/mL. (**h**) Heat map showing the ELISA binding of neutralizing serum IgG antibodies purified from ePTC donors to recombinant mutant and truncated gp120 proteins. Color values are proportional to the reactivity levels measured as % of binding compared to wild-type gp120. (**i**) Competition heatmap showing the relative ELISA binding of neutralizing serum IgG antibodies purified from ePTC donors to HIV-1 gp120 in presence of selected HIV-1 bNAbs (% as compared to serum IgGs alone). Darker blue colors indicate stronger inhibition while white indicates no competition. Asterisk in (a), (b), (d) indicates ePTC rebounders. Source data are provided as a Source Data file.

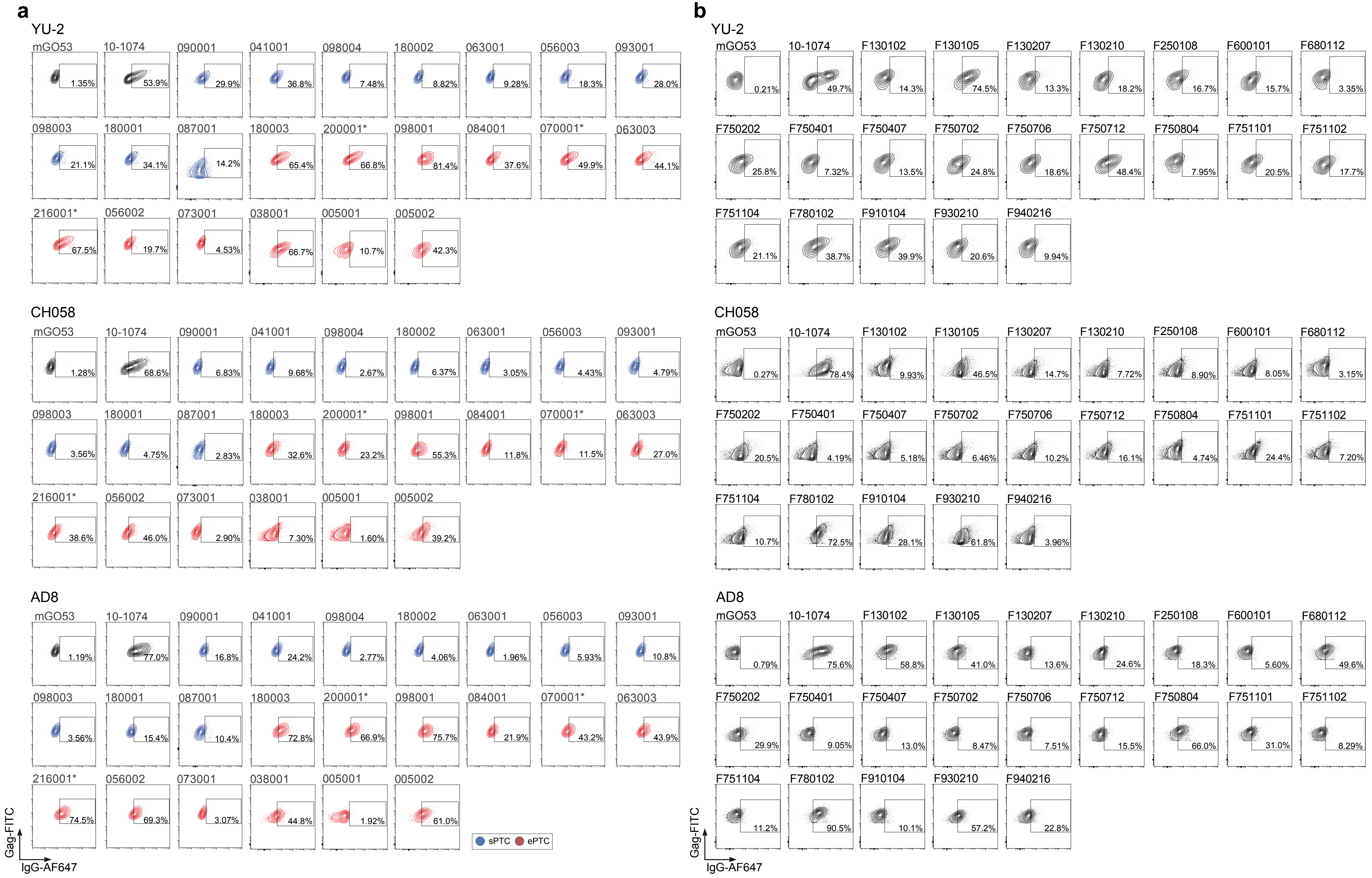

Supplementary Fig. 3. Serum IgG antibody binding to HIV-1-infected target cells in PTC and PTNC. (**a**) Representative flow cytograms showing the binding of serum IgG antibodies purified from PTC to Gag^{+}CEM.NKR-CCR5 target cells infected with AD8, YU2 and CH058 viruses. mGO53 and 10-1074 are negative and positive control antibodies, respectively. Percentages of IgG^{+}Gag^{+} cells are indicated in the gates. (**b**) Same as in (a) but for PTNC donors. Asterisks indicate ePTC rebounders.

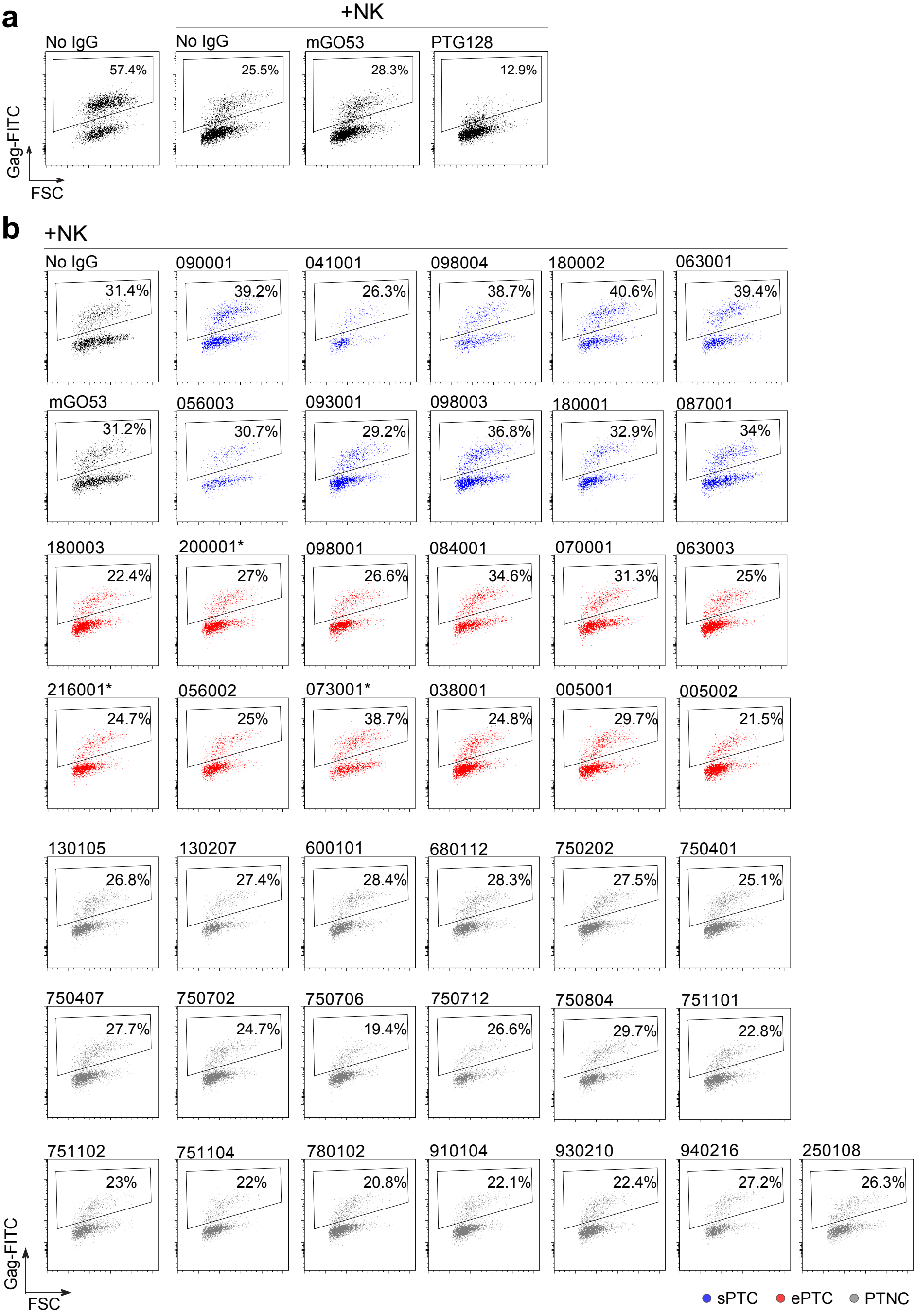

Supplementary Fig. 4. ADCC activity of purified serum IgG antibodies from PTC and PTNC. (**a**) Representative flow cytograms showing the % of Gag^{+} CEM.NKR-CCR5 cells infected with the CH058 T/F virus following a 4 h incubation period with or without human primary NK cells in the presence or absence of negative (mGO53) and positive (PGT128) control IgG antibodies. (**b**) Same as in (a) but with purified IgGs from PTC and PTNC donors (at a final concentration of 50 µg/ml). Asterisks indicate ePTC rebounders.

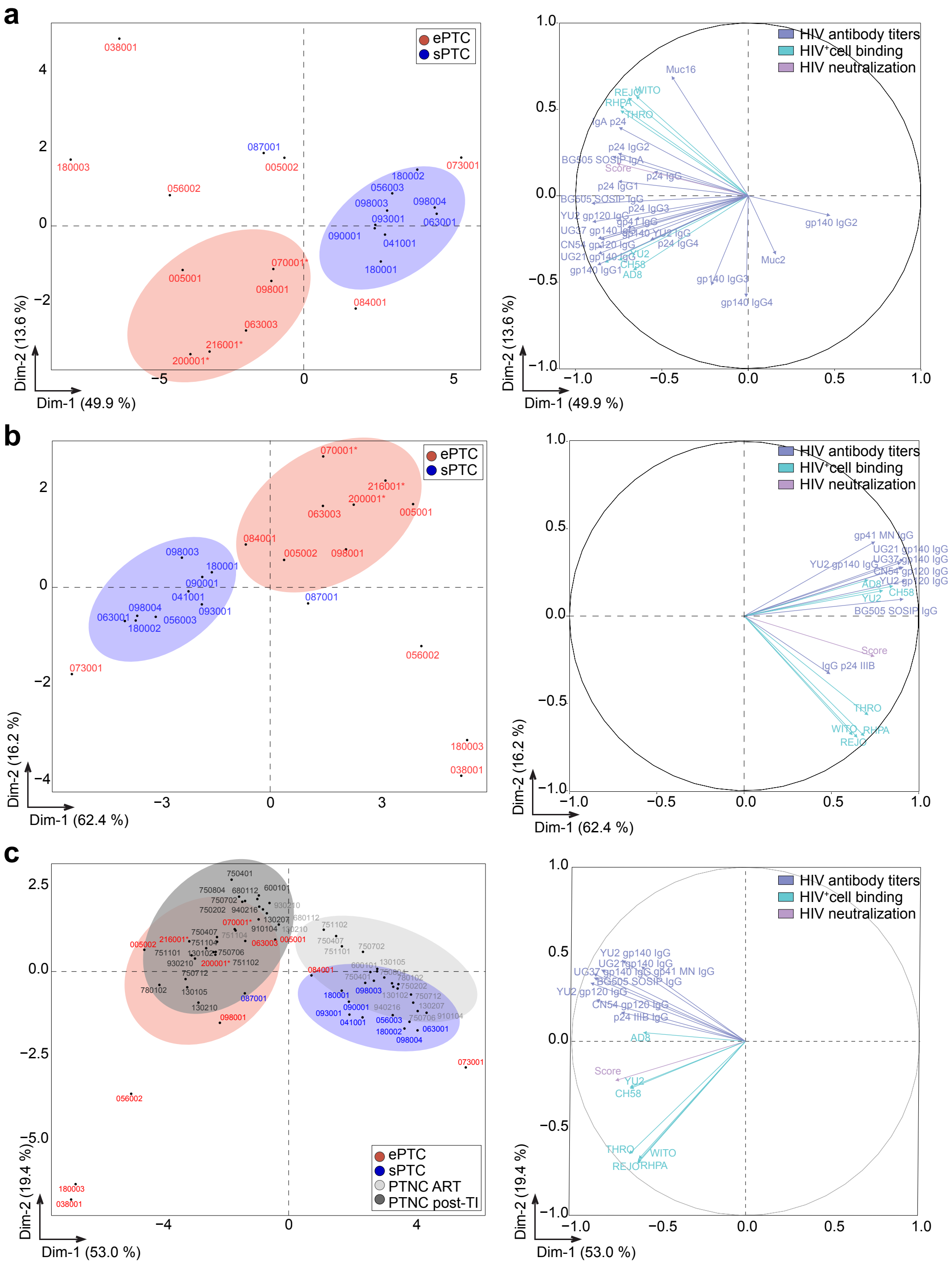

Supplementary Fig. 5. PCA of serum antibody parameters in PTC and PTNC. (**a**) Individual (left) and variable (right) PCA diagrams generated using a total 28 serological parameters measured in sPTC (red, n=12) and ePTC (blue, n=10) as shown in Fig. 2c. (**b**) Same as in (a) but with a total of 16 selected serological parameters. (**c**) Same as in (b) but including data from PTNC (n=20) under and after ART (light and dark grey, respectively). Asterisks indicate ePTC rebounders.

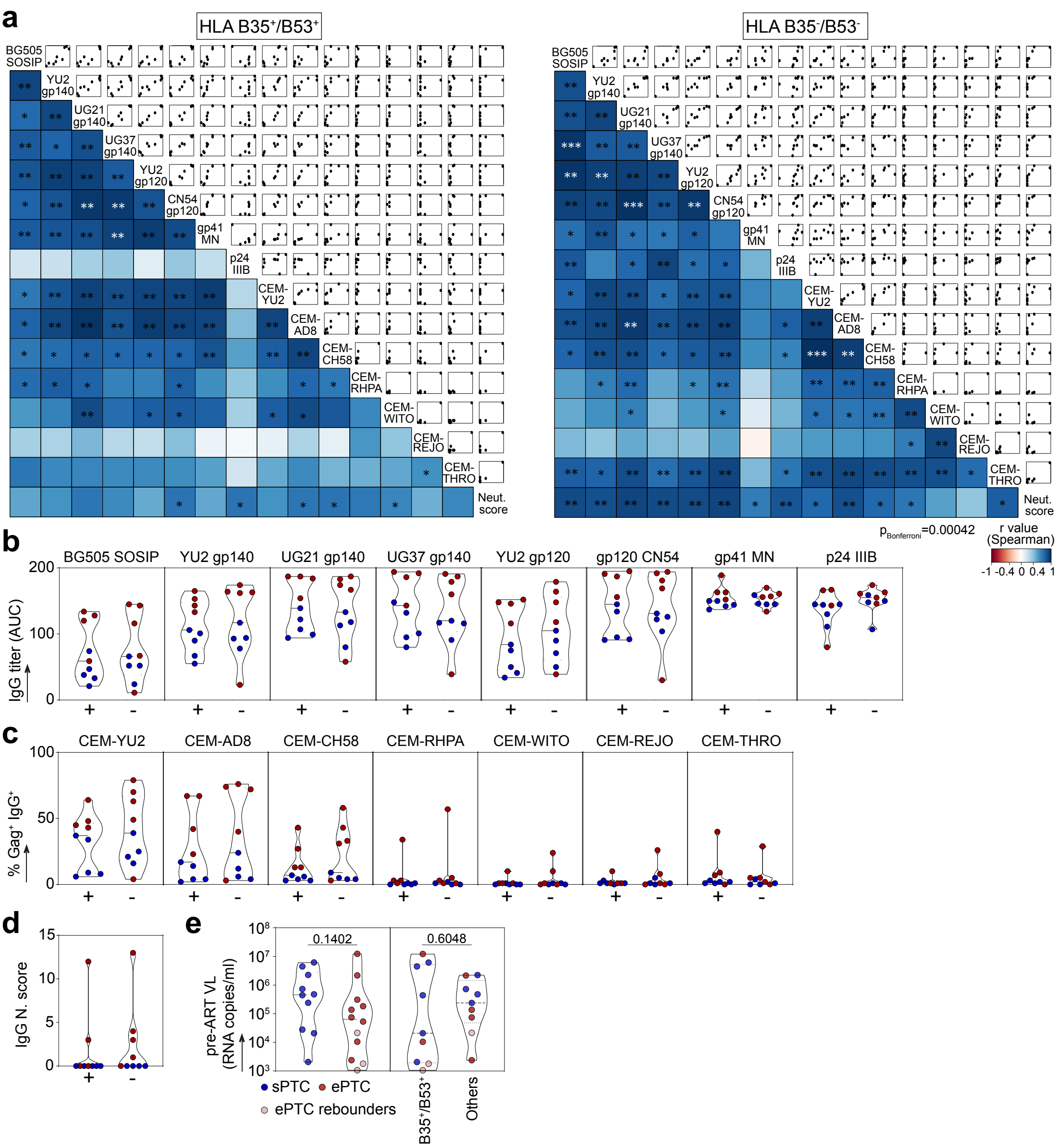

Supplementary Fig. 6. HLA class I B35 and B53 allele expression and serum antibody parameters in PTC. (**a**) Correlograms showing the correlation analyses of the humoral immune parameters measured in PTC carrying HLA B35 or B53 allele (left) and $B35^-/B53^-$ (right) including IgG antibody titers, binding to HIV-1-infected cells and neutralizing activity. For each pair of compared parameters, scatter plots are shown on top and two-sided Spearman correlation coefficients (color coded) with their corresponding *p* values at the bottom. Asterisks correspond to unadjusted *p* values. ***p<0.0001, **p<0.01, *p<0.05. *p* values below the Bonferroni-corrected significance threshold are highlighted in white. Detailed correlation results are presented in Supplementary data 1. (**b**) Violin plots comparing the serum anti-Env and anti-p24 IgG titers between PTC carrying B35/53 HLA alleles (+, n=9) or not (-, n=9). Red and blue dots indicate ePTC and sPTC, respectively. (**c**) Same as in (b) but for the IgG binding to HIV-1-infected CEM.NKR-CCR5 cells. (**d**) Same as in (b) but for the *in vitro* IgG seroneutralizing activity. (**e**) Violin plots comparing the pre-ART VL between sPTC (blue, n=10) and ePTC (red, n=12) (left), and between donors carrying (n=9) or not B35/53 (n=9) HLA alleles (right). ePTC rebounders are shown in light red. 2-tailed Mann-Whitney test was performed and the *p* values are indicated over the horizontal bars on top. Source data are provided as a Source Data file.

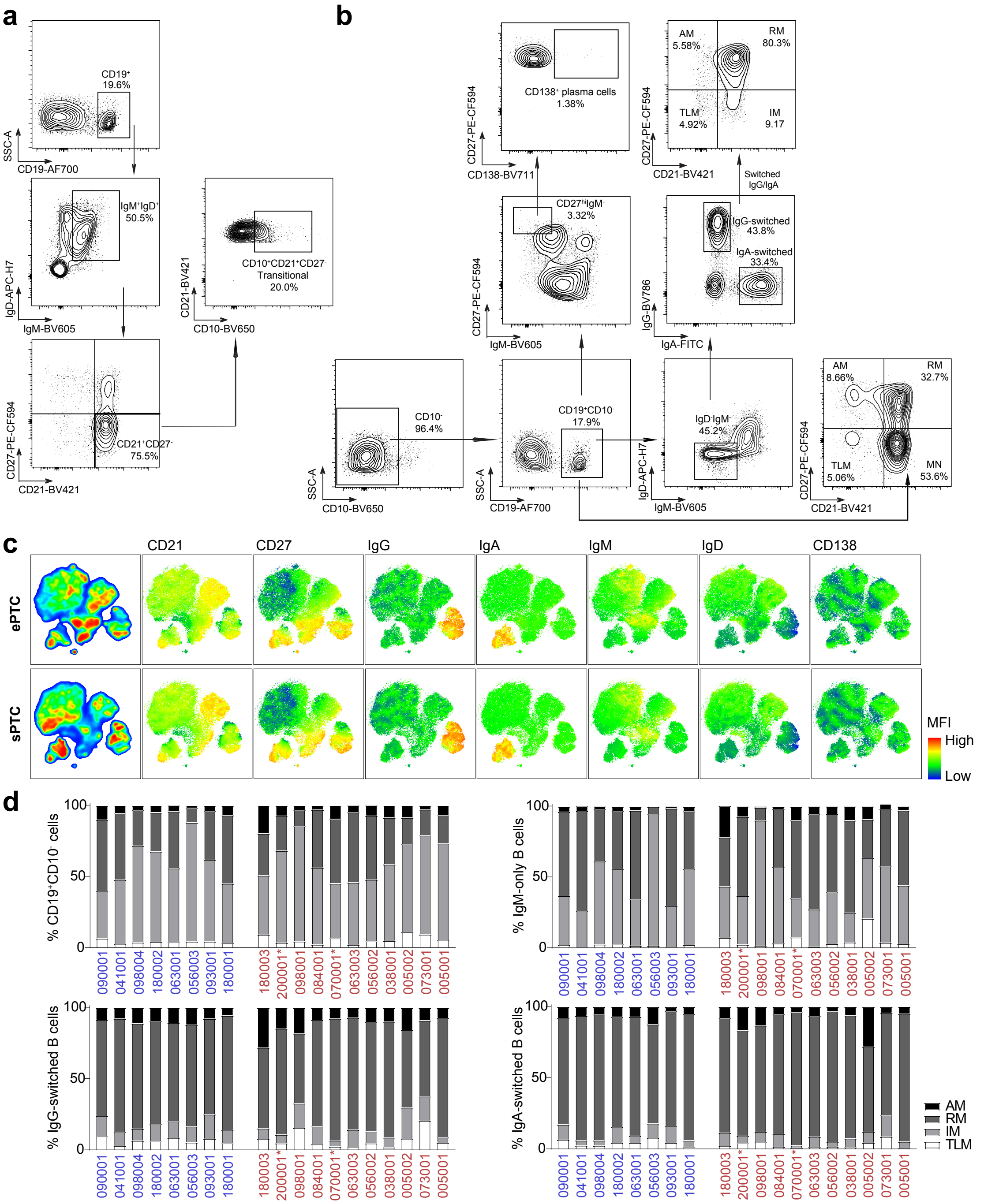

Supplementary Fig. 7. B-cell immunophenotyping in PTC. (**a**) Flow cytograms showing the gating strategy used to determine the frequency of circulating blood total and transitional B cells. (**b**) Same as (a) but for mature naïve B cells and memory B-cell subsets. (**c**) t-SNE-derived statistic heatmaps showing the median fluorescence intensity (MFI) of the indicated surface markers. (**d**) Bar diagrams comparing the individual distribution of memory B-cell subsets in total CD10$^-$CD19$^+$, IgM-only, and class-switched (IgG$^+$ and IgA$^+$) B cells between sPTC (blue, n=8) or ePTC (red, n=11). ePTC rebounders are indicated with an asterisk. MN, mature naïve; AM, activated memory; RM, resting memory; IM, intermediate memory; TLM, tissue-like memory. Source data are provided as a Source Data file.

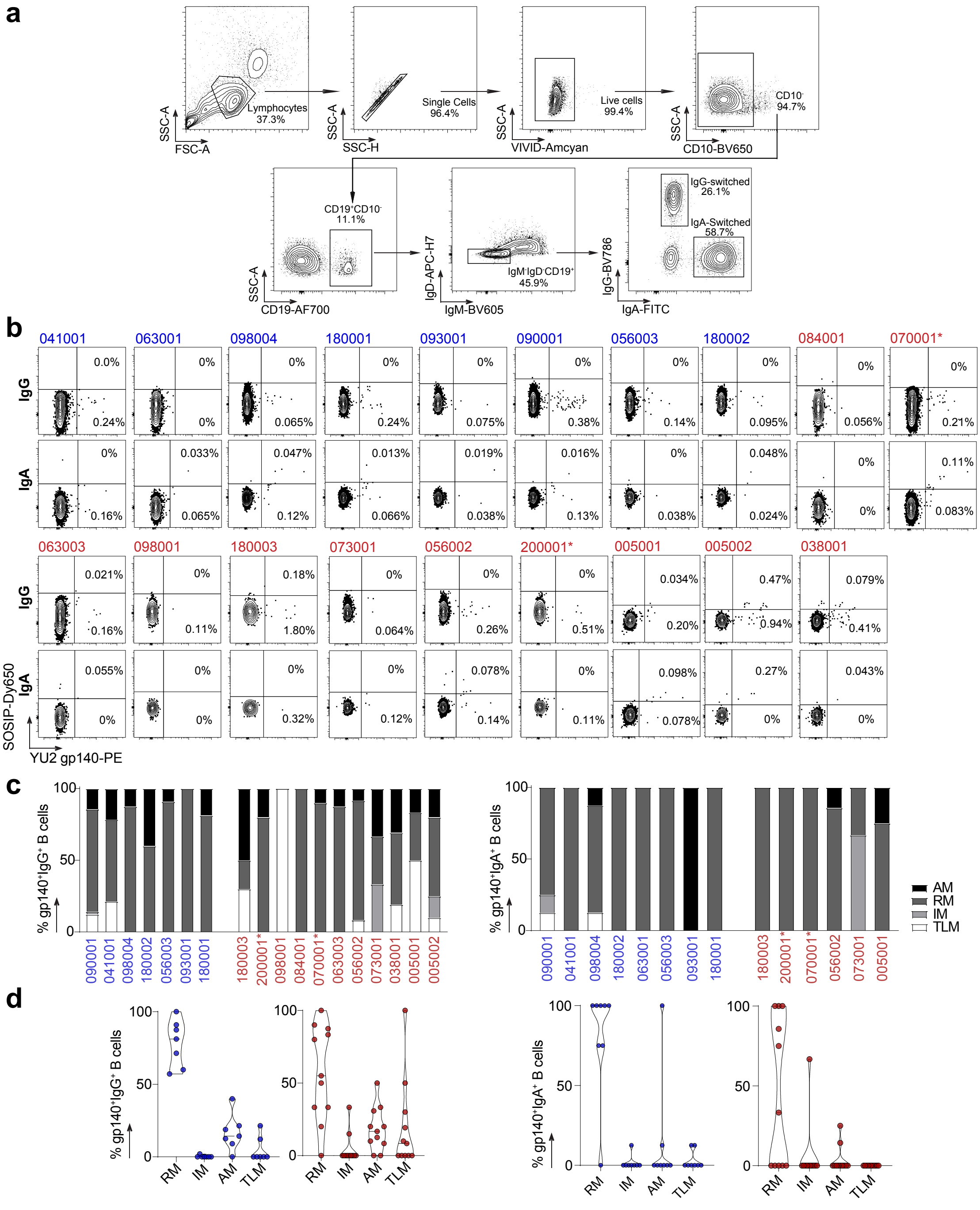

Supplementary Fig. 8. Blood HIV-1 Env-reactive memory B cells in PTC. (**a**) Flow cytograms showing the gating strategy used to identify circulating blood IgG+ and IgA+ memory B cells in PTC. (**b**) Flow cytograms showing the staining of IgG+ and IgA+ memory B cells (as shown in (a)) with fluorescently-labelled YU2 gp140-F and BG505 SOSIP trimers in sPTC (blue) and ePTC (red). Percentages of gp140+ and gp140+SOSIP+ IgG+, and IgA+ B cells are indicated in the specific gates. (**c**) Bar diagrams comparing the individual distribution of memory B-cell subsets in gp140-F-specific IgG+ and IgA+ memory B cells between sPTC (blue, IgG n=7, IgA n=8) and ePTC (red, IgG n=11, IgA n=6). (**d**) Violin plots comparing the frequency of gp140-specific IgG+ and IgA+ memory B cells in each memory B-cell subset between sPTC (blue, IgG n=7, IgA n=8) and ePTC (red, IgG n=11, IgA n=6). ePTC rebounders are indicated with an asterisk. Source data are provided as a Source Data file.

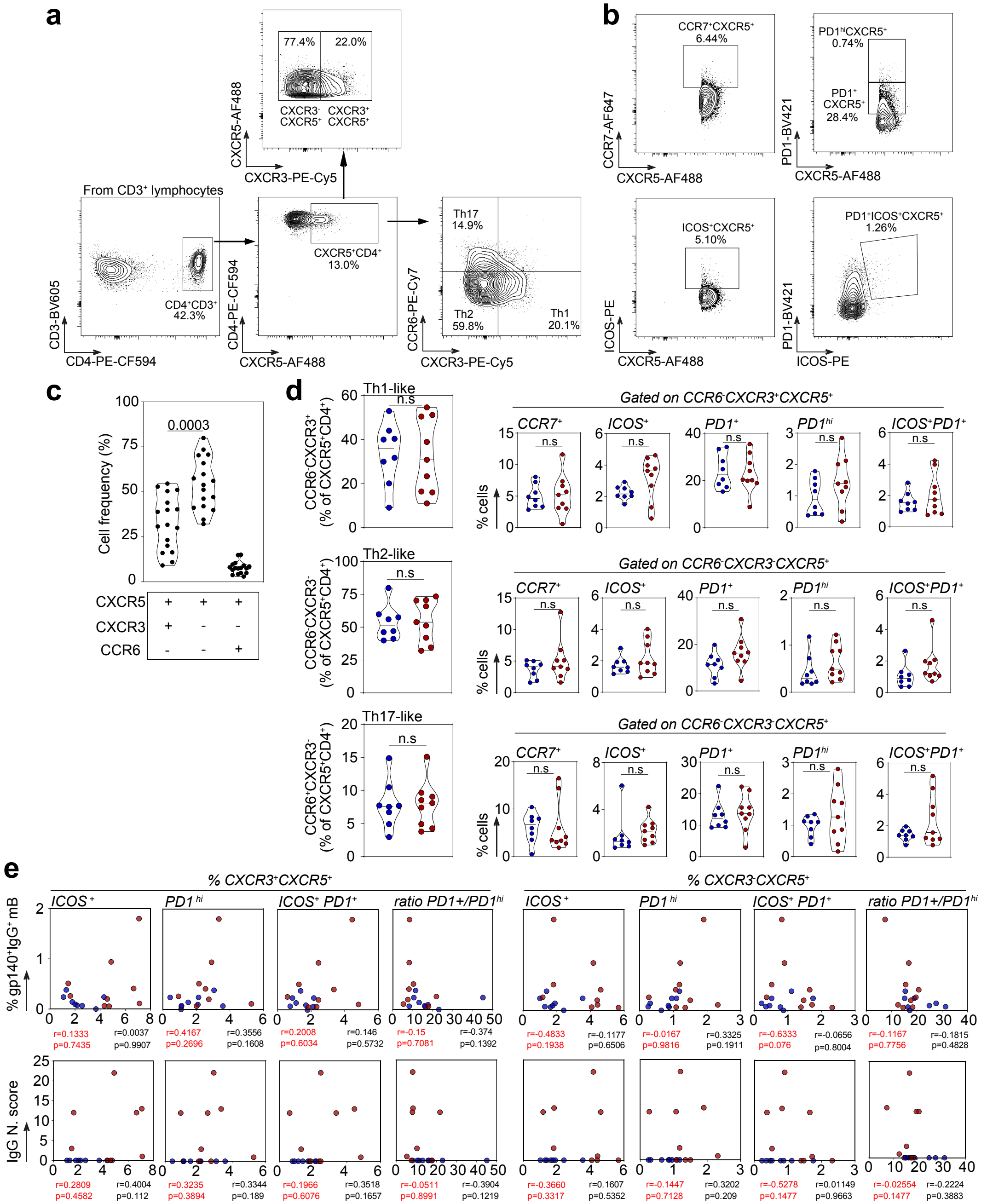

Supplementary Fig. 9. cTfh-cell immunophenotyping in PTC. (**a**) Flow cytograms showing the gating strategy used to determine the frequency of circulating follicular helper T (cTfh)-cell subsets. (**b**) Same as in (a) but for activated *vs* quiescent cTfh cells gated on the different cTfh-cell subsets. (**c**) Violin plots showing the proportion of Th1 ($CCR6^-CXCR3^+$), Th2 ($CCR6^-CXCR3^-$) and Th17 ($CCR6^+CXCR3^-$) -like cTfh cells in PTC (n=17). + or - indicates the presence or absence of the indicated markers, respectively. (**d**) Violin plots comparing between ePTC (red, n=9) and sPTC (blue, n=8), the frequency of total Th1, Th2 and Th17-like cTfh cells (left), and of $CCR7^+$, $ICOS^+$, $PD1^+$, $PD1^{hi}$, and $ICOS^+PD1^+$ among these cTfh-cell subsets (right). Groups were compared using 2-tailed Mann-Whitney test in (c-d). ns, not significant ($p > 0.05$). (**e**) Correlation plots comparing the % of $gp140^+IgG^+$ memory B cells and IgG seroneutralization score (N. score) with the % of indicated blood cTfh-cell subsets. Statistical values for all PTC (n=17) and ePTC (n=9) only are indicated in black and red, respectively. Source data are provided as a Source Data file.
