# Supplementary material for: Transient viral exposure drives functionally-coordinated humoral immune responses in HIV-1 post-treatment controllers
Source: Nat Commun. 2022 Apr 11;13:1944. doi: 10.1038/s41467-022-29511-1 (PMC9001681; doi:10.1038/s41467-022-29511-1)
Supplement: Supplementary file 3 — Description of Additional Supplementary Files [file 41467_2022_29511_MOESM3_ESM.pdf]

Description of Additional Supplementary Files

Supplementary Data 1: Statistics of multiparametric analyses
