# Supplementary material for: Transient viral exposure drives functionally-coordinated humoral immune responses in HIV-1 post-treatment controllers
Source: Nat Commun. 2022 Apr 11;13:1944. doi: 10.1038/s41467-022-29511-1 (PMC9001681; doi:10.1038/s41467-022-29511-1)
Supplement: Supplementary file 5 — Reporting summary [file 41467_2022_29511_MOESM5_ESM.pdf]

nature portfolio

Corresponding author(s): Hugo Mouquet

Last updated by author(s): Feb 21, 2022

Reporting Summary

Nature Portfolio wishes to improve the reproducibility of the work that we publish. This form provides structure for consistency and transparency in reporting. For further information on Nature Portfolio policies, see our Editorial Policies and the Editorial Policy Checklist.

Statistics

For all statistical analyses, confirm that the following items are present in the figure legend, table legend, main text, or Methods section.

n/a	Confirmed	
☐	☒	The exact sample size (*n*) for each experimental group/condition, given as a discrete number and unit of measurement
☐	☒	A statement on whether measurements were taken from distinct samples or whether the same sample was measured repeatedly
☐	☒	The statistical test(s) used AND whether they are one- or two-sided<br>*Only common tests should be described solely by name; describe more complex techniques in the Methods section.*
☐	☒	A description of all covariates tested
☐	☒	A description of any assumptions or corrections, such as tests of normality and adjustment for multiple comparisons
☐	☒	A full description of the statistical parameters including central tendency (e.g. means) or other basic estimates (e.g. regression coefficient) AND variation (e.g. standard deviation) or associated estimates of uncertainty (e.g. confidence intervals)
☐	☒	For null hypothesis testing, the test statistic (e.g. *F*, *t*, *r*) with confidence intervals, effect sizes, degrees of freedom and *P* value noted<br>*Give P values as exact values whenever suitable.*
☒	☐	For Bayesian analysis, information on the choice of priors and Markov chain Monte Carlo settings
☒	☐	For hierarchical and complex designs, identification of the appropriate level for tests and full reporting of outcomes
☐	☒	Estimates of effect sizes (e.g. Cohen's *d*, Pearson's *r*), indicating how they were calculated

Our web collection on statistics for biologists contains articles on many of the points above.

Software and code

Policy information about availability of computer code

Data collection	ELISA: Sunrise™ microplate absorbance reader (Tecan Männedorf, Switzerland). Magellan software v7.2 (TECAN)<br>IFA: Axio Imager 2 (Zeiss) fluorescence microscope. ZEN imaging software (Zen 2.0 blue version, Zeiss)<br>Neutralization: Wallac 1420 VICTOR2™ microplate reader (Perkin Elmer). Wallac 1420 Workstation<br>Flow cytometry opsonization/ADCC assay: Attune™Nxt instrument (Life Technologies). Attune NxT Software v3.1.2<br>Flow cytometry immunophenotyping: BD LSRFortessa™ instrument (BD Biosciences). FACS Diva v8.0.1 (BD)
Data analysis	GraphPad Prism v6.0a and v9.2.0 (GraphPad)<br>R Studio Server (v1.4.1103). factoextra package (v1.0.7, https://CRAN.R-project.org/package=factoextra)<br>FlowJo (v10.6, FlowJo LLC, Ashland, OR)<br>Qlucore Omics Explorer software v3.7 (Qlucore AB)<br>ZEN imaging software (Zen 2.0 blue version, Zeiss)

For manuscripts utilizing custom algorithms or software that are central to the research but not yet described in published literature, software must be made available to editors and reviewers. We strongly encourage code deposition in a community repository (e.g. GitHub). See the Nature Portfolio guidelines for submitting code & software for further information.

Data

Policy information about availability of data

All manuscripts must include a data availability statement. This statement should provide the following information, where applicable:

- Accession codes, unique identifiers, or web links for publicly available datasets
- A description of any restrictions on data availability
- For clinical datasets or third party data, please ensure that the statement adheres to our policy

Source data are provided in the manuscript.
All statistical values calculated from the multiparameter correlations are also provided.

Field-specific reporting

Please select the one below that is the best fit for your research. If you are not sure, read the appropriate sections before making your selection.

☒ Life sciences ☐ Behavioural & social sciences ☐ Ecological, evolutionary & environmental sciences

For a reference copy of the document with all sections, see nature.com/documents/nr-reporting-summary-flat.pdf

Life sciences study design

All studies must disclose on these points even when the disclosure is negative.

Sample size	This is a descriptive and exploratory study of a rare population of HIV-infected individuals. Determination of sample size could not be applied as we included and analyzed all samples possibly available.
Data exclusions	No data exclusions were performed.
Replication	All immunoserological experiments were performed in duplicate or triplicate, in at least two independent determinations. ADCC experiments were performed in duplicate using NK effector cells from 8 different healthy donors.<br>Depending on sample availability, flow cytometry immunophenotyping experiments were reproduced 1-2 times.<br>All experiments were reproduced successfully.
Randomization	Randomization does not apply because the population of study were specifically post-treatment Controllers (PTC), individuals which are part of the Viro-Immunological Sustained COntrol after Treatment Interruption (VISCONTI) Study of the ANRS CO21 CODEX cohort.<br>Post-treatment non-controllers (PTNC) were selected to match PTC according to ART initiation (51.5 [26-100] days post-infection), treatment duration (3.8 [0.48-11.17] years) and HLA B35/B53 distribution. PTNC are part of the ANRS CO6 PRIMO cohort.<br>Details can be found in Methods and Supplementary table 1.
Blinding	Blinding was not applied because: (i) this was not an interventional study, and (ii) individuals were selected according to defined criteria (see aforementioned).

Reporting for specific materials, systems and methods

We require information from authors about some types of materials, experimental systems and methods used in many studies. Here, indicate whether each material, system or method listed is relevant to your study. If you are not sure if a list item applies to your research, read the appropriate section before selecting a response.

Materials & experimental systems

n/a	Involved in the study	
☐	☒	Antibodies
☐	☒	Eukaryotic cell lines
☒	☐	Palaeontology and archaeology
☒	☐	Animals and other organisms
☐	☒	Human research participants
☒	☐	Clinical data
☒	☐	Dual use research of concern

Methods

n/a	Involved in the study	
☒	☐	ChIP-seq
☐	☒	Flow cytometry
☒	☐	MRI-based neuroimaging

Antibodies

Antibodies used	ELISAs - Secondary conjugated antibodies used for revelation: Polyclonal goat peroxydase-conjugated anti-human IgG

(#109-035-098) and anti-human IgA (#109-035-011) [Immunology Jackson ImmunoReseach]. The antibodies were used at a final concentration of 0.8 µg/ml.
Flow cytometry_B-cell antibody panel: CD19 A700 (HIB19, 1:50 dilution), CD21 BV421 (B-ly45, 1:50 dilution), CD27 PE-CF594 (M-T271, 1:100 dilution), CD10 BV650 (HI10a, 1:50 dilution), CD138 BV711 (MI15, 1:62.5 dilution), IgM BV605 (G20-127, 1:50 dilution), IgG BV786 (G18-145, 1:50 dilution), IgD APC-H7 (IA6-2, 1:100 dilution) (BD Biosciences), IgA FITC (IS11-8E10, 1:62.5 dilution) (Miltenyi Biotec).
Flow cytometry_T-cell antibody panel: CD3 BV605 (SK7, 1:50 dilution), CD4 PE-CF594 (RPA-T4, 1:50 dilution), CD185/CXCR5 AF-488 (RF8B2, 1:25 dilution), CD183/CXCR3 PE-Cy™5 (1C6/CXCR3, 1:12.5 dilution), CD196/CCR6 PE-Cy™7 (11A9, 1:50 dilution), CD197/CCR7 AF647 (3D12, 1:50 dilution) (BD Biosciences), CD279/PD1 BV421 (EH12.2H7, 1:50 dilution) (BioLegend), and CD278/ICOS PE (ISA-3, 1:50 dilution) (Thermo Fisher Scientific).
CEM-NKR binding and ADCC assays: anti-human IgG1 (H+L) Alexa Fluor 647 (1:400 dilution; #A-21445, Life Technologies); anti-HIV-1 core FITC KC57 (1:500 dilution; #6604665, Beckman Coulter)

Validation

The antibodies are commercially available and the reactivity validated against the appropriate species, as reported in manufacture´s website, technical datasheets and previous studies.

CD19 A700 (clone HIB19, #557921, BD Biosciences): According to manufacturer´s website the antibody is routinely tested in flow cytometry. Flow cytometric analysis of CD19 expression on human peripheral blood lymphocytes is provided in the website and technical datasheet. Supporting references are also provided.
CD21 BV421 (clone B-ly45, #562966, BD Biosciences): According to manufacturer´s website the antibody is routinely tested in flow cytometry. Flow cytometric analysis of CD21 expression on human peripheral blood lymphocytes is provided in the website and technical datasheet. Supporting references are also provided.
CD27 PE-CF594 (clone M-T271, #562297, BD Biosciences): According to manufacturer´s website the antibody is routinely tested in flow cytometry. Flow cytometric analysis of CD27 expression on human peripheral blood lymphocytes is provided in the website and technical datasheet. Supporting references are also provided.
CD10 BV650 (clone HI10a, #563734, BD Biosciences): According to manufacturer´s website the antibody is routinely tested in flow cytometry. Flow cytometric analysis of CD10 expression on human REH (Acute B cell leukemia, ATCC CRL-8283) cells is provided in the website and technical datasheet. Supporting references are also provided.
CD138 BV711 (clone MI15, #563184, BD Biosciences): According to manufacturer´s website the antibody is routinely tested in flow cytometry. Flow cytometric analysis of CD138 expression on U266 cells is provided in the website and technical datasheet.
IgM BV605 (clone G20-127, #562977, BD Biosciences): According to manufacturer´s website the antibody is routinely tested in flow cytometry. A two color flow cytometric analysis of IgM expression on human peripheral blood lymphocytes is provided in the website and technical datasheet. Supporting references are also provided.
IgG BV786 (clone G18-145, #564230, BD Biosciences): According to manufacturer´s website the antibody is routinely tested in flow cytometry. A two color flow cytometric analysis of IgG expression on human peripheral blood lymphocytes is provided in the website and technical datasheet. Supporting references are also provided.
IgD APC-H7 (clone IA6-2, #561305, BD Biosciences): According to manufacturer´s website the antibody is routinely tested in flow cytometry. Flow cytometric analysis of IgD expression on human peripheral blood lymphocytes is provided in the website and technical datasheet. Supporting references are also provided.
IgA FITC (clone IS11-8E10, #130-114-001, Miltenyi Biotec): According to manufacturer´s website, the antibody is suited for flow cytometry. Flow cytometric analysis of IgA expression on human peripheral blood mononuclear cells is provided in the website and technical datasheet. Extended validations on epitope specificity, sensitivity and fixation data are also provided. Supporting references are also provided.
CD3 BV605 (clone SK7, #563219, BD Biosciences): According to manufacturer´s website the antibody is routinely tested in flow cytometry. Flow cytometric analysis of CD3 expression on human peripheral blood mononuclear cells is provided in the website and technical datasheet. Supporting references are also provided.
CD4 PE-CF594 (clone RPA-T4, #562281, BD Biosciences): According to manufacturer´s website the antibody is routinely tested in flow cytometry. Flow cytometric analysis of CD4 expression on human peripheral blood lymphocytes is provided in the website and technical datasheet. Supporting references are also provided.
CD185/CXCR5 AF-488 (clone RF8B2, #558112, BD Biosciences): According to manufacturer´s website the antibody is routinely tested in flow cytometry. Flow cytometric analysis of CXCR5 (CD185) expression on human peripheral blood lymphocytes is provided in the website and technical datasheet. Supporting references are also provided.
CD183/CXCR3 PE-Cy™5 (clone 1C6/CXCR3, #551128, BD Biosciences) According to manufacturer´s website the antibody is routinely tested in flow cytometry. Flow cytometric analysis of CD183 expression on human peripheral blood lymphocytes is provided in the website and technical datasheet. Supporting references are also provided.
CD196/CCR6 PE-Cy™7 (clone 11A9, #560620, BD Biosciences) According to manufacturer´s website the antibody is routinely tested in flow cytometry. Flow cytometric analysis of CD196 expression on human lysed whole blood is provided in the website and technical datasheet. Supporting references are also provided.
CD197/CCR7 AF647 (clone 3D12, #557734, BD Biosciences) According to manufacturer´s website the antibody is routinely tested in flow cytometry. Flow cytometric analysis of CCR7 (CD197) expression on CD4 and CD8-positive human peripheral lymphocytes is provided in the website and technical datasheet. Supporting references are also provided.
CD279/PD1 BV421 (clone EH12.2H7, #329920, Biolegend) According to manufacturer´s website each lot of the antibody is routinely tested in flow cytometry. A two-color flow cytometric analysis of CD279 (PD1) expression on human peripheral blood lymphocytes is provided in the website and technical datasheet. Supporting references are also provided.
CD278/ICOS PE (clone ISA-3, 12-9948-41, Thermo Fisher Scientific) According to manufacturer´s website the antibody was validated by flow cytometric analysis by measuring the expression of CD278 (ICOS) on 3-day unstimulated and 3-day anti-CD3/CD28-stimulated human peripheral blood cells. Supporting references are also provided.

Eukaryotic cell lines

Policy information about cell lines

Cell line source(s)	HEK-293T cells (CRL-11268™, ATCC).<br>Freestyle™ 293-F cells (R79007, Thermo Fisher Scientific).<br>TZM-bl cells (#8129, NIH AIDS Reagent Program).<br>CEM.NKR-CCR5+ cells (#4376, NIH AIDS Reagent Program).
Authentication	Authentication was based on morphology, growth and expected behaviour and functionality.
Mycoplasma contamination	Mycoplasma contamination not tested.
Commonly misidentified lines (See ICLAC register)	Misidentified lines were not used.

Human research participants

Policy information about studies involving human research participants

Population characteristics

The Post-Treatment Controllers (PTC) involved in the study presented an average age of 48 [20-71] years old, and a M/F gender distribution of 14 / 8. PTC are part of the Viro-Immunological Sustained COntrol after Treatment Interruption (VISCONTI) Study of the ANRS CO21 CODEX cohort. Some of the PTC included in the study have been previously described (1). The post-Treatment Non-Controllers (PTNC) described in the study presented an average age of 36 [25-53] years old, and a M/F gender distribution of 19 / 2. PTNC are participants in the ANRS CO6 PRIMO cohort (2), who initiated ART during primary infection but experienced post-TI viral rebound. The HIV-1 status and clinico-virological information on the donors of the PTC VISCONTI and PTNC PRIMO cohorts are detailed in the Supplementary table 1.

1. A. Sáez-Cirión, C. Bacchus, L. Hocqueloux, V. Avettand-Fenoel, I. Girault, C. Lecuroux, V. Potard, P. Versmisse, A. Melard, T. Prazuck, B. Descours, J. Guergnon, J. P. Viard, F. Boufassa, O. Lambotte, C. Goujard, L. Meyer, D. Costagliola, A. Venet, G. Pancino, B. Autran, C. Rouzioux, Post-Treatment HIV-1 Controllers with a Long-Term Virological Remission after the Interruption of Early Initiated Antiretroviral Therapy ANRS VISCONTI Study. PLoS Pathogens 9, (2013)

2. S. Novelli, C. Lecuroux, V. Avettand-Fenoel, R. Seng, A. Essat, P. Morlat, J. P. Viard, C. Rouzioux, L. Meyer, C. Goujard, Long-term Therapeutic Impact of the Timing of Antiretroviral Therapy in Patients Diagnosed With Primary Human Immunodeficiency Virus Type 1 Infection. Clin Infect Dis 66, 1519-1527 (2018).

Recruitment

The recruitment criteria of the subjects in this study were based exclusively in clinico-virological characteristics without any additional bias.
The inclusion criteria of PTC (n=22) were: (1) to present a viral load >2000 RNA copies/ml at ART initiation; (2) to be under ART during at least 12 months with suppressed viremia; and (3) to maintain a viral load below 400 RNA copies/ml for more than one year after treatment discontinuation with at least two viral load determinations during this period.
The selection of PTNC (n=21) was based on matched characteristics with PTC: HLAB35/53 prevalence, ART initiation (51.5 [26-100] days post-infection) and treatment duration (3.8 [0.48-11.17] years). PTNC rebounded on average within the year post-treatment discontinuation.
The clinico-virological information on PTC VISCONTI and PTNC PRIMO donors are detailed in the Supplementary table 1.

Ethics oversight

Samples were obtained in accordance with and after ethical approval from all the French legislation and regulation authorities. The clinical research protocol received approval from ethical committee (comité de protection des personnes, CPP) Ile-de-France VII. All donors gave written consent to participate in this study, and data were collected under pseudo-anonymized conditions using subject coding.

Note that full information on the approval of the study protocol must also be provided in the manuscript.

Flow Cytometry

Plots

Confirm that:

- [x] The axis labels state the marker and fluorochrome used (e.g. CD4-FITC).
- [x] The axis scales are clearly visible. Include numbers along axes only for bottom left plot of group (a 'group' is an analysis of identical markers).
- [x] All plots are contour plots with outliers or pseudocolor plots.
- [x] A numerical value for number of cells or percentage (with statistics) is provided.

Methodology

Sample preparation

- Immunophenotyping of lymphocyte populations was performed on cryopreserved Peripheral Blood Mononuclear Cells Cells (PBMC). Cells were first stained using LIVE/DEAD fixable dead cell stain kit (405 nm excitation) (Molecular Probes, Thermo Fisher Scientific) to exclude dead cells. B and cTfh lymphocyte subsets were analyzed using two different

fluorescently labeled antibody cocktails. For B-cell phenotyping, PBMC were first incubated with biotinylated YU2 gp140 and DyLight650-coupled BG505 SOSIP Env trimers for 30 min at 4°C. Cells were then washed once with 1% FBS-PBS (FACS buffer), and incubated for 30 min at 4°C with the following antibodies of the B-cell staining panel: CD19 A700 (HIB19), CD21 BV421 (B-ly4), CD27 PE-CF594 (M-T271), CD10 BV650 (HI10a), CD138 BV711 (MI15), IgM BV605 (G20-127), IgG BV786 (G18-145), IgD APC-H7 (IA6-2) (BD Biosciences), IgA FITC (IS11-8E10, Miltenyi Biotec), and streptavidin R-PE conjugate (Invitrogen, Thermo Fisher Scientific). The cTfh antibody panel included: CD3 BV605 (SK7), CD4 PE-CF594 (RPA-T4), CD185/CXCR5 AF-488 (RF8B2), CD183/CXCR3 PE-Cy™5 (1C6/CXCR3), CD196/CCR6 PE-Cy™7 (11A9), CD197/CCR7 AF647 (3D12) (BD Biosciences), CD279/PD1 BV421 (EH12.2H7, BioLegend), and CD278/ICOS PE (ISA-3, Thermo Fisher Scientific). Finally, cells were washed and resuspended in FACS buffer, and then fixed in 1% paraformaldehyde-PBS.
- CEM.NKR-CCR5 cells (NIH AIDS Reagent Program) were infected with inocula of selected viruses, and adjusted to achieve 10-30% of Gag+ cells at 48 h post infection. Infected cells were incubated with purified serum IgG (50 µg/ml) in PBS containing 0.5% BSA and 2 mM EDTA, for 30 min at 37°C, washed and incubated with AF647-conjugated anti-human IgG antibody (1:400 dilution; Life technologies) for 30 min at 4°C. Cells were then fixed with 4% paraformaldehyde and stained for intracellular Gag (KC57-FITC 1/500 in PBS/1%BSA/Azide/saponin 0.05%).

Instrument

- BD LSRFortessa™ instrument (BD Biosciences), for PBMC staining.
- Attune Nxt instrument (Life Technologies), for CEM.NKr-CCR5 cell staining.

Software

FlowJo software (v10.6, FlowJo LLC)

Cell population abundance

Cell sorting was not performed.

Gating strategy

Lymphocytes were gated based on FSC-A/SSC-A, followed by single cell check based on SSC-A/SSC-H. Live cells were then gated based on a LIVE/DEAD fixable dead cell stain kit (405 nm excitation, Molecular Probes, ThermoFicher Scientific). B and T lymphocyte subsets were then analyzed using the specified antibody cocktails following the gating strategies shown in Supplementary Figures 7, 8 and 9.

☒ Tick this box to confirm that a figure exemplifying the gating strategy is provided in the Supplementary Information.
